# *Aspergillus luchuensis*, an Endophyte Fungus from the Metal Hyperaccumulator Plant *Prosopis laevigata*, Promotes Its Growth and Increases Metal Translocation

**DOI:** 10.3390/plants12061338

**Published:** 2023-03-16

**Authors:** Efraín Tovar-Sánchez, Cynthia Margarita Concepción-Acosta, Ayixon Sánchez-Reyes, Ricardo Sánchez-Cruz, Jorge Luis Folch-Mallol, Patricia Mussali-Galante

**Affiliations:** 1 Centro de Investigación en Biodiversidad y Conservación, Universidad Autónoma del Estado de Morelos, Av. Universidad 1001, Col. Chamilpa, CP, Cuernavaca 62209, Morelos, Mexico; 2 Centro de Investigación en Biotecnología, Universidad Autónoma del Estado de Morelos, Av. Universidad 1001, Col. Chamilpa, CP, Cuernavaca 62209, Morelos, Mexico,; 3 Investigador por México, CONACyT, Institute of Biotechnology, Universidad Nacional Autónoma de México, Ave. Universidad 2001, Col. Chamilpa, Cuernavaca 62210, Morelos, Mexico

**Keywords:** heavy metals, phytoremediation, *Prosopis laevigata*, metal translocation, copper, *Aspergillus*

## Abstract

Heavy metal pollution is a worldwide environmental and human health problem. *Prosopis laevigata* is a hyperaccumulator legume that bioaccumulates Pb, Cu and Zn. With interest in designing phytoremediation strategies for sites contaminated with heavy metals, we isolated and characterized endophytic fungi from the roots of *P. laevigata* growing on mine tailings located in Morelos, Mexico. Ten endophytic isolates were selected by morphological discrimination and a preliminary minimum inhibitory concentration was determined for zinc, lead and copper. A novel strain of *Aspergillus* closest to *Aspergillus luchuensis* was determined to be a metallophile and presented a marked tolerance to high concentrations of Cu, Zn and Pb, so it was further investigated for removal of metals and promotion of plant growth under greenhouse conditions. The control substrate with fungi promoted larger size characters in *P. laevigata* individuals in comparison with the other treatments, demonstrating that *A. luchuensis* strain C7 is a growth-promoting agent for *P. laevigata* individuals. The fungus favors the translocation of metals from roots to leaves in *P. laevigata*, promoting an increased Cu translocation. This new *A. luchuensis* strain showed endophytic character and plant growth-promotion activity, high metal tolerance, and an ability to increase copper translocation. We propose it as a novel, effective and sustainable bioremediation strategy for copper-polluted soils.

## 1. Introduction

Heavy metal pollution is one of the most complicated pollution problems to eliminate, since these elements accumulate in the environment and in organisms and are not biodegradable like other xenobiotic compounds such as pesticides or hydrocarbons. Chemical and physical techniques have been used with some success to remediate polluted sites with heavy metals, but they are costly and leave behind byproducts that must be dealt with [1].

Many heavy metals in certain concentrations are essential for life, since they are cofactors that are necessary for enzymatic catalysis; for example, most hydrolases use Zn, Mg, and other metals to perform their activity [2,3]. Other enzymes use a variety of metals; for example, nitrogenase has an Fe, Mo, Co or V metallocluster to reduce N_2_ [4]. Oxydoreductases use copper ions to oxidize their substrates [5], and many more examples could be given. However, when heavy metal levels rise above certain concentrations, they become very toxic to almost all life forms. This toxicity is due to several mechanisms that involve the generation of reactive oxygen species (ROS), leading to genotoxicity or impairment of enzyme activity by binding either to the active site or to other parts of proteins [1,6], among others.

Bioremediation, in contrast to chemical or physical treatments, offers a sustainable and cost-effective way to remove heavy metals from soils. In this sense, the most widely used technique for bioremediation of heavy metals is phytoremediation [7,8,9,10].

However, increasing attention is being focused on microorganisms as heavy metal remediators. Both bacteria and fungi have been reported to have the ability to cope with high heavy metal concentrations due to their ability to detoxify such elements [11]. Most of the work using microbes to remove or decrease the toxicity of heavy metals has been focused on bacteria [11]. Mycoremediation of heavy metals is a strategy that is still to be explored more carefully, although there are some works that deal with this issue [12,13,14]. Also, some scientific approaches have undertaken the study of a binomial approach using plants and fungal interactions [15,16]. Fungi may have some advantages over bacteria when treating heavy metal-polluted waters or soils. Firstly, their hyphal growth produces a mycelium that exposes a large area of contact with the polluted site. Fungi have a complex cell wall mainly made from chitin and beta1,3-glucans [17]—molecules with polar or charged groups (polyols or positive charges in the N-acetylglucosamine polymer that forms chitin) that allow interactions with various redox states of heavy metals, immobilizing them by adsorption. Another important advantage of fungi is that they possess an endomembrane system (vacuoles, endoplasmic reticulum, lysosomes, etc.) that can compartmentalize heavy metals, isolating them from the cytoplasm [18]. They also produce and secrete metabolites such as organic acids that can chelate many ions, or intracellular molecules such as glutathione and metallothioneins—proteins that can sequester heavy metals [19,20]. Also, mushroom residues have been used as an amendment that promotes plant growth and heavy metal phytostabilization [21]. Finally, many fungi establish long-lasting symbioses with plants, and this interaction enhances plant development, greater host plant biomass, and tolerance to abiotic and biotic stresses, resulting in higher HM bioaccumulation levels. Most of the studies of these relationships have been studied in arbuscular mycorrhizae [12] and *Trichoderma* spp. [22,23,24], although other fungal species have also been reported [25]. Nevertheless, even the studies using arbuscular mycorrhizae and *Trichoderma* spp. are still underexplored, and the mechanisms that enhance phytoremediation of heavy metals in endophytic relationships are still poorly understood [26]. *Prosopis laevigata* (Fabaceae) is a tree species that develops naturally in mine tailings and has been reported to bioaccumulate Pb, Cu and Zn [27]. The *Aspergillus* strain isolated from *P. laevigata* roots was characterized due to (a) its capacity to tolerate high HM concentrations, which would ensure its survival in heavily metal-polluted soils; (b) its plant growth-promotion trait that could benefit *P. leviagata* breeding by allowing transplantation earlier than non-inoculated plants in polluted sites and (c) the possibility that the plant–fungal interaction could enhance heavy metal translocation to the shoot, which is the main mechanism of heavy metal detoxification by hyperaccumulator plants [27]. Hence, the aims of the present work were to analyze the fungal endophytic community of the hyperaccumulator plant *Prosopis laevigata*, and a very promising *Aspergillus* strain isolated from *P. laevigata* roots was identified at the molecular level and evaluated regarding its HM translocation potential in *P. laevigata* individuals. 

## 2. Results

### 2.1. Isolation and Screening of Endophytic Fungi from P. laevigata

**Figure 1:**
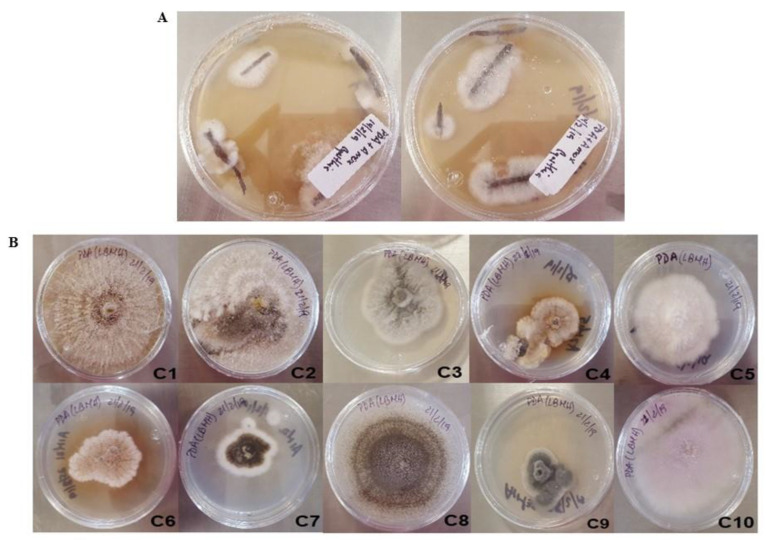
Fungal isolates from *Prosopis laevigata* roots from a mine tailing. (**A**) Mycelia emerging from the disinfected roots. (**B**) Isolated morphotypes after visual screening.

From surface-sterilized roots, about 20 different fungal morphotypes were recovered. All the isolates sporulated, so the spores were collected, and single colonies were recovered after three monosporic passes to ensure a homogeneous isolate. Visual inspection of the isolates allowed the selection of 10 clearly different morphotypes (Figure 1), to avoid siblings.

### 2.2. Preliminary Screening for Metal Tolerance of the Isolates from P. laevigata Roots

Since the plants collected for the isolation of endophytes were growing in a metal- polluted location, we tested the tolerance to the three most abundant metals on the site: Cu, Zn and Pb. Two extreme concentrations of these metals were used for a preliminary screening for metal tolerance, according to previous results for each metal’s toxicity [28] (Table 1).

From this screening we selected six isolates that showed medium to good growth even in the highest concentrations of each metal, except for Zn, which was very toxic to most of the strains. For example, strain C9 was discarded, since it grew poorly in all the tested concentrations, while strain C5 showed good growth, even in the highest concentration of Zn. Thus, strains C1, C2, C5, C7, C8 and C10 were selected to continue with their characterization, and the Minimum Inhibitory Concentration of each metal was tested for these strains.

### 2.3. Characterization of Growth Rate and MIC for Selected Isolates from P. laevigata Roots

**Figure 2:**
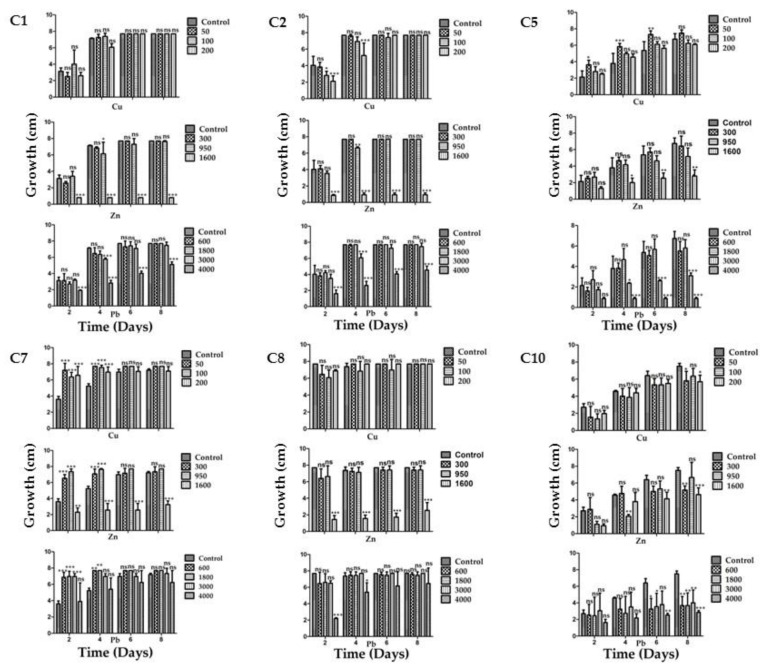
Minimum inhibitory concentration for the six most tolerant strains isolated from *P. laevigata* roots in each of the three metals tested. The strain’s code is depicted at the upper left corner for each group of three graphs, which includes one for each metal (Cu, Zn and Pb). * = *p* < 0.05, ** = *p* < 0.01, *** = *p* < 0.001, ns = no significant difference.

Each strain was grown individually in PDA medium supplemented with different amounts of Cu, Zn or Pb. Further on, three mixtures with different concentrations of the three metals were tested to emulate the conditions in the mine tailing. Figure 2 shows the growth of each of the six strains with each metal tested.

Strains C1 and C2 showed a similar behavior, although their morphotype was different; they were extremely tolerant for all the metals tested except for Zn and Pb at the highest concentrations (1600 ppm and 4000 ppm, respectively). Otherwise, they grew at the same rate as in the control media without metals. Nonetheless, they could still grow well in 4000 ppm of lead, with Zn being the most toxic element when used at 1600 ppm, where no growth was observed. However, they could still grow in the control medium when 900 ppm of Zn was used, which indicates a good level of tolerance.

Strain C5 proved to be a metallophile for Cu since it grew better in 50 ppm than in the control without metals (Figure 2). However, in general this strain showed a lower growth rate than strains C1 and C2 in all cases. A distinctive feature of strain C5 is that, in contrast with strains C1 and C2, it showed a significant growth rate in 1600 ppm of Zn, but it was sensitive to 3000 and 4000 ppm of Pb.

Strain C7 performed very well, growing in any of the three metals; it grew better than in the control without metals, showing its metallophilic characteristic for the three metals tested, except in 1600 ppm of Zn (Figure 2). Nevertheless, it could also grow well in the latter condition.

Strain C8 presented a similar phenotype to that of strains C1 and C2, with the exception that this strain showed growth in 1600 ppm pf Zn and a good level of tolerance in 900 ppm of Zn.

Finally, strain C10 showed the slowest growth rate when compared to the other strains (even without metals). It was tolerant to Cu since it grew as well as in the media without this metal. For Zn it still showed a good tolerance since it could grow even at 1600 ppm, although less than in the control medium without metals, and showed the highest sensitivity to Pb compared to the other strains (Figure 2).

**Figure 3:**
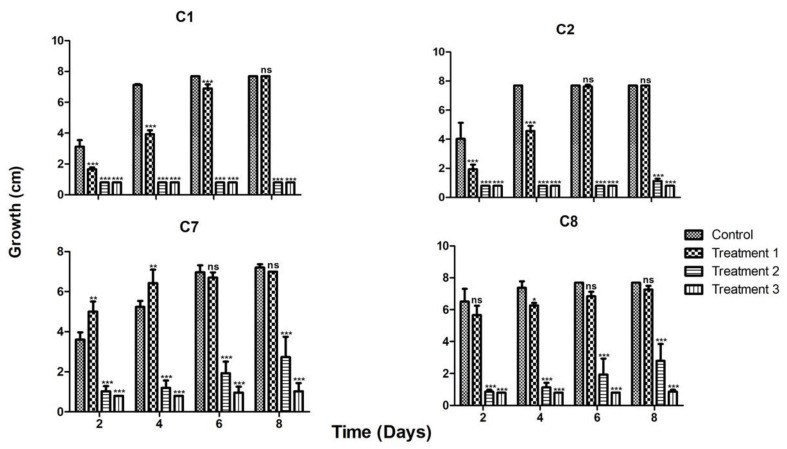
Behavior of the four most tolerant strains in the presence of the three metals in three treatments. The strain’s code is depicted up at the center for each group of the four graphs, growth is depicted by bars in the figures for the following treatments. Treatment 1 contained: 50 ppm Cu, 300 ppm Zn and 600 ppm Pb; treatment 2 contained: 100 ppm Cu, 950 ppm Zn and 1800 ppm Pb; and treatment 3 contained: 200 ppm Cu, 1600 ppm Zn and 4000 ppm Pb. * = *p* < 0.05, ** = *p* < 0.01, *** = *p* < 0.001, ns = no significant difference.

When the strains were grown in the presence of the three previously tested metals, strains C1 and C2 again showed a similar behavior in the three treatments, being very sensitive to treatments 2 and 3, which contained higher Zn concentrations (900 and 1600 ppm, respectively; Figure 3). Strains C7 and C8 behaved similarly amongst them, but in contrast to strains C1 and C2 they could withstand treatment 2, although at a slower growth rate. Strain C7 could even grow slightly in treatment 3 (Figure 3). This is in accordance with the observed results for the strains tested in media with single metals.

This set of experiments indicated that the most tolerant strain for all the metals tested was C7, hence we decided to identify this strain and test it for its characteristics of growth promotion of *P. laevigata* plants and whether this strain could aid the plant regarding metal uptake and translocation.

### 2.4. Identification and Molecular Analysis of Strain C7

**Figure 4:**
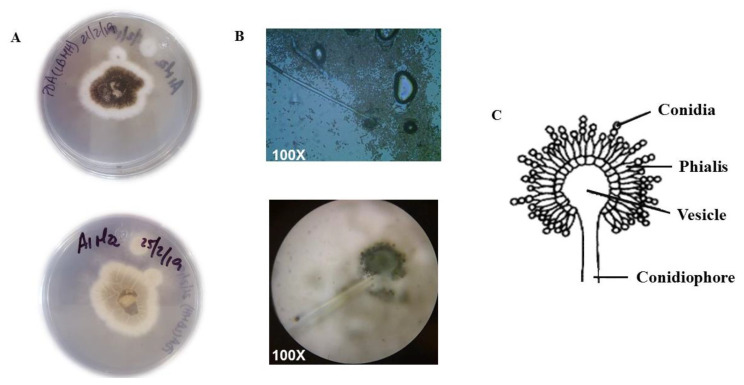
Macroscopic and microscopic appearance of strain C7. (**A**) PDA Petri dishes showing the mycelium from above (upper dish) and from below (lower dish). (**B**) Microscopic observation of the conidiophores and conidia of strain C7. (**C**) Drawing representing the asexual reproductive apparatus of *Aspegillus* species.

Macroscopic observation of the fungus showed irregular-shaped colonies with black spores in the center. The mycelium was white and cottony, with a convex curvature. On the back part of the Petri dish the mycelium appeared white with a smooth basal surface. Microscopic observation of strain C7 showed conidiophores consistent with the genus *Aspergillus* (Figure 4).

Since the group Aspergilli is very complex, we obtained a representation of the genomic composition for strain C7 by ONT long-read sequencing to classify this strain as best we could. Although we did not obtain a sufficient sequencing depth to assemble a complete genome, we were able to infer from the reads the regions homologous to 28S, calmodulin and β-tubulin markers. The Blast search against the NCBI database verified that the closest local phylogenetic neighbors of strain C7 belong to the genus *Aspergillus* (Table 2) and seems to be more distant from *Penicillium*.

**Figure 5:**
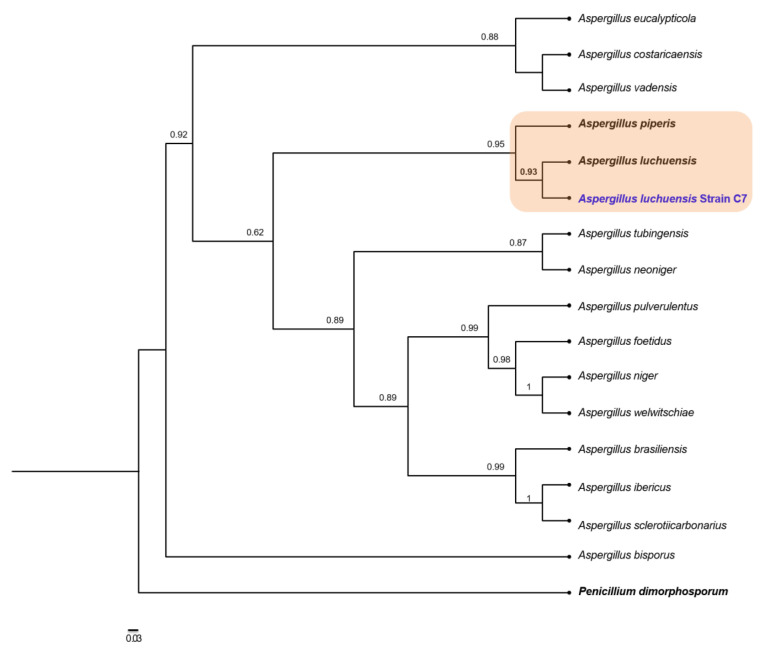
Maximum likelihood phylogeny based on concatenated alignments of 28S, calmodulin and β-tubulin sequences. Approximate likelihood-ratio test (aLRT) for branches is shown in nodes. *Penicillium dimorphosporum* was used as outgroup. The scaled bar under the tree indicates substitutions per site.

With microscopic observations and local alignment evidence converging toward the relevance of the *Aspergillus* genus for the C7 strain, we inferred a phylogeny with the concatenated 28S, calmodulin and β-tubulin markers (Figure 5). C7 clustered in a clade with *A. luchuensis* and *A. piperis* with highly supported aLRT values. The aLRT around the branches for *A. luchuensis* and C7 was supported by 0.93, suggesting a likely correct arrangement. Finally, we wanted to evaluate the genomic coherence of strain C7 with *A. luchuensis* and *A. piperis*. Hence, we estimated the genomic distance and the containment of the four representative closest fungal genomes vs. the set of long reads obtained in this study for C7 (Table 3). C7 is highly coherent with *A. luchuensis* and *A. piperis* as observed before in the phylogenetic reconstruction, although it shares a greater number of genomic objects and containment with *A. luchuensis*; also, they share less genomic distance (*D* = 0.037). None of these indexes are “universal” for the classification of fungi, but they do constitute evidence that can help establish species-specific limits and to identify overall genomic transition signatures. According to our results, we cannot rule out the hypothesis that strain C7 is consistent with *A. luchuensis*. First, we did not detect a relevant phylogenetic transition between these two contexts (as it is clear for *A. piperis* that clusters in a clearly divergent branch of C7 and *A. luchuensis*); second, genomically, a relevant transition between C7 and *A. luchuensis* is also not observed: they share containment indices of 0.98 (close to the threshold of 0.99) [29], which indicates that the *A. luchuensis* genome is significantly contained in the raw C7 long-reads, and 715 genomic objects are identified as common between both contexts. All the results indicate that C7 belongs to the species *A. luchuensis*.

### 2.5. Plant Growth Promotion of A. luchuensis C7 on P. laevigata in Substrates with or without Metals

**Figure 6:**
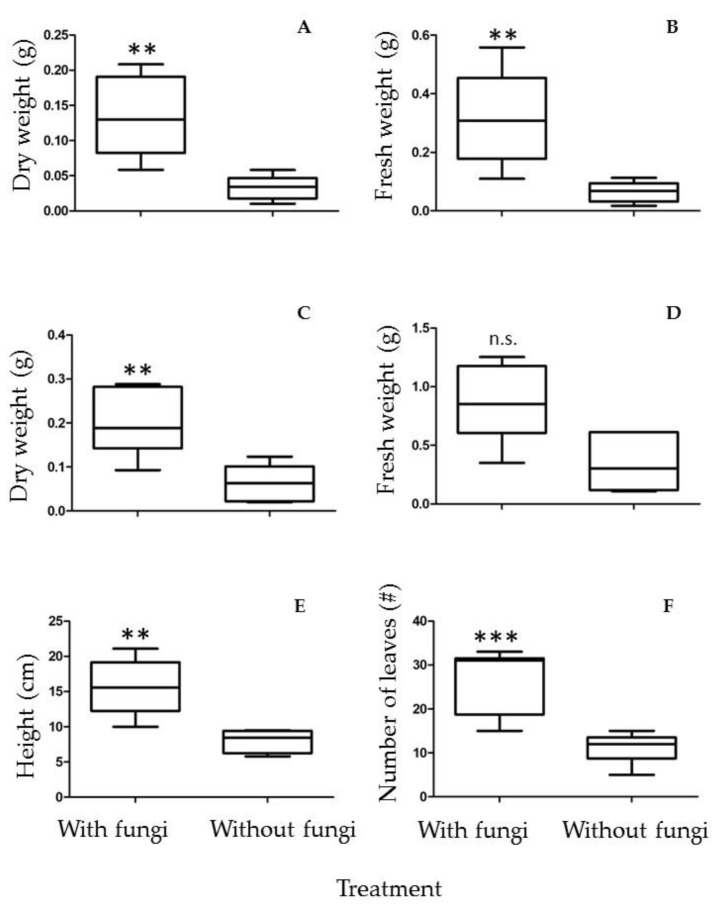
Plant growth-promoting activity of *A. luchuensis* C7 on *P. laevigata* plants grown on control substrate. (**A**) Leaf dry weight, (**B**) Leaf fresh weight, (**C**) Root dry weight, (**D**) Root fresh weight, (**E**) Plant height, (**F**) Number of leaves. n.s. = no significant difference, ** *p* < 0.01; *** *p* < 0.001.

An important feature in plant–fungal interactions is whether the fungus can increase plant growth, thus conferring an advantage in plant propagation and sustainability in substrates where nutrients are scarce or non-bioavailable. *P. laevigata* plants were tested in both substrates, one coming from the mine tailing and a control substrate collected from an unpolluted location (see Materials and Methods). When tested in the mine tailing substrate, no significant difference in dry weight, fresh weight, leaf number and height of the plants was observed after two months of growth in the presence or absence of the fungus. Nevertheless, no detrimental effect in the presence of the fungus was observed with the plant parameters tested. However, in the control substrate a clear positive and statistically significant effect of the fungus on fresh weight, dry weight, number of leaves and plant height was recorded after two months of growth (Figure 6).

**Figure 7:**
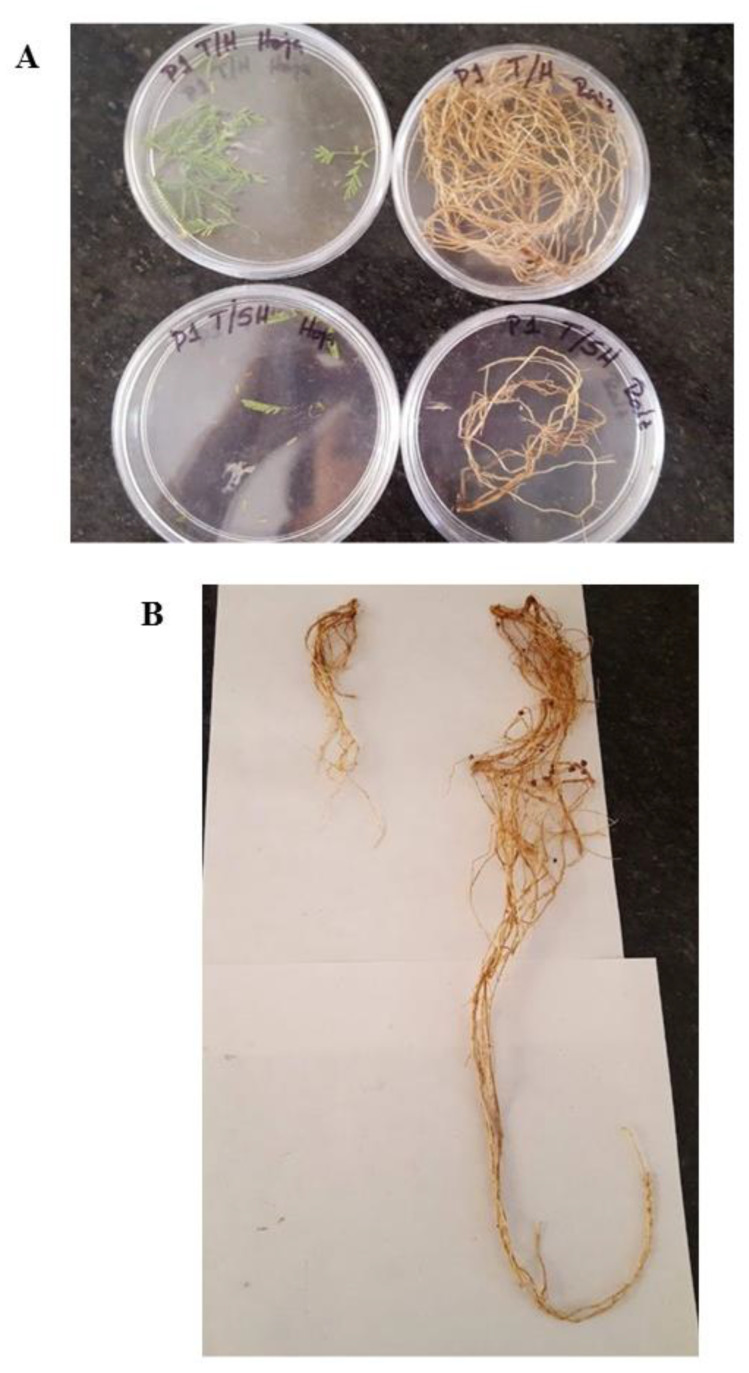
Representative images of a *P. laevigata* specimen grown in control substrate in the presence or absence of the strain *A. luchuensis* C7. (**A**) Foliar (left) and root (right) biomass collected from a *P. laevigata* in the presence (upper images) or absence (lower images) of *A. luchuensis* C7. (**B**) Root length of a representative *P. laevigata* plant in the presence (upper images) or absence (lower image) of strain *A. luchuensis* C7.

Figure 7 shows representative images of *P. leviagata* plants grown in the control substrate in the presence or absence of *A. luchuensis* C7.

### 2.6. Influence of Heavy Metal Exposure on Size Characters in P. laevigata Individuals

**Figure 8:**
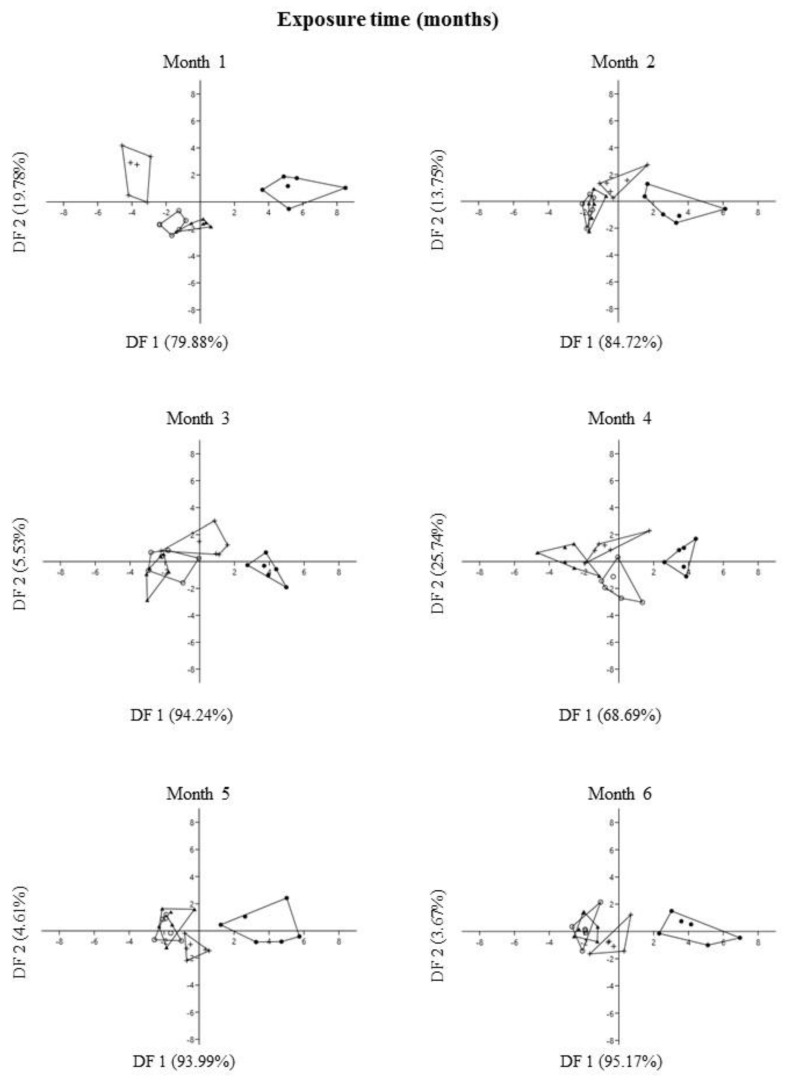
Plot of DF1 vs. DF2 extracted through Discriminant Function Analysis of each size character of *Prosopis laevigata* under greenhouse conditions. Each point is a two-dimensional (axis 1 and axis 2) representation of size character ordination. Treatments: control with fungi (●), control without fungi (+), tailing with fungi (○), tailing without fungi (▲).

The results of DFA showed that the control substrate with and without fungi promoted changes in size characters of *P. laevigata* individuals. Size characters of *P. laevigata* were grouped into two well-defined groups, belonging to control substrate with and without fungi, which were separated based on size characters, while individuals growing on tailing substrate with and without fungi were overlapped, regardless of exposure time (Figure 8).

In general, DFA explained 98.30 ± 1.98 (mean ± s.d.) of the variation of the total data from DF1 and DF2 for size characters during six months of exposure (Supplementary Material Table S1, Figure 8). The variables that most contributed positively to the DF1 ordination were dry foliar biomass followed by wet foliar biomass. In contrast, the variables that most contributed negatively were dry foliar biomass = wet leaf biomass > aerial plant height = dry root biomass. On the other hand, the variables that most contributed positively to the DF2 ordination were dry leaf biomass > wet leaf biomass, while the variables that most contributed negatively were wet leaf biomass > dry root biomass = wet root biomass = dry leaf biomass (Supplementary Material Table S1, Figure 8).

### 2.7. Effect of Treatments on Size Characters in P. laevigata Individuals

In general, one-way ANOVA detected a significant effect of all treatments (control with fungi, control without fungi, tailing with fungi and tailing without fungi) on all size characters in *P. laevigata* individuals, independently of exposure time, except for aerial plant height at month 4. Tukey post hoc analysis showed that the individuals growing on control substrate with fungi had consistently greater size values of all characters in comparison with the other treatments (Table 4).

### 2.8. Relationship between Exposure Time and Size Characters in P. laevigata Individuals

The statistically significant percentage of the relationships found between exposure time (six months) and the size characters in *P. laevigata* individuals showed the next pattern: control substrate with fungi (100%) > control substrate without fungi (66.7%) > tailing substrate with fungi (33.3%) = tailing substrate without fungi. Also, all the statistically significant relationships were positive, meaning that as the exposure time increased the size characters were also increased (Supplementary Material Tables S2 and S3).

### 2.9. Heavy Metal Bioaccumulation in P. laevigata Individuals along Exposure Times

*P. laevigata* individuals growing on substrate with fungi registered a negative and significant relationship between Cu bioaccumulation in root and exposure time. In contrast, a positive and significant relationship between Cu bioaccumulation in leaves and exposure time was detected (Supplementary Material Table S4). In other words, as exposure time increases, *P. laevigata* individuals accumulate less Cu in the roots and more in the leaves (see heavy metal concentration in Supplementary Material Table S2).

### 2.10. Translocation Coefficient in P. laevigata Individuals

In general, *P. laevigata* individuals growing on substrate with fungi registered higher mean translocation coefficient values in comparison with individuals growing on substrate without fungi. Mean translocation values for Pb, Cu and Zn registered the next pattern: Pb > Cu > Zn (Table 5).

A positive and significant relationship was recorded between exposure time and Cu translocation levels in individuals growing on tailing substrate with fungi. In contrast, no relationship was detected between exposure time and translocation levels for the rest of the treatments and metals (Table 5).

## 3. Discussion

### 3.1. Molecular Analysis and Metal Tolerance of the Isolates from P. laevigata Roots

Binomial approaches using microbe–plant interactions have recently drawn the attention of many research groups, since several works have shown that microorganisms associated with plants can also be relevant for bioremediation strategies [30], especially those regarding heavy metal pollution [10,31,32]. However, there are only a few reports that describe fungal communities associated with hyperaccumulator plants, and most of them focus only on Arbuscular Mycorhizae [33].

In this work, the isolation of twenty endophytic fungal strains from *P. laevigata* roots, growing in mine tailings, were preliminary classified into ten morphotypes. These results indicate that even in a toxic environment like heavy metal-polluted soils, fungi show a considerable diversity, probably through interactions with hyperaccumulator plants. Ten isolates were further characterized, and the six most metal-tolerant strains were studied. Strain C5 was a metallophile for Cu, but not for Zn or Pb, indicating that different metal elements are not handled by a unique mechanism and the fungus discriminates between them. In contrast, strain C7 showed this metallophilic characteristic for the three tested metals since it grew better in the presence of metals than in their absence. Strain C8 did not show this characteristic (Figure 2 and Figure 3). Nonetheless, all six fungi were able to grow in the presence of up to 200 ppm of Cu, 950 ppm of Zn and, except for C5, up to 4000 ppm of Pb.

When the three metals were added in the same medium at three different concentrations, strains C1 and C2 could only achieve growth in the treatment with the lowest metal concentration (50 ppm Cu, 300 ppm Zn and 600 ppm Pb), while strains C7 and C8 were also able to grow in treatment 2 (100 ppm Cu, 950 ppm Zn and 1800 ppm Pb). A noticeable difference between the latter strains is that C7 maintained its metallophilic character, while strain C8 grew better without metals. The fact that strain C7 was an endophyte and showed a metallophilic characteristic could enhance the bioremediation potential of *P. laevigata*. The metallophilic character of fungi depends on the species genome when they possess genes for metal transport to the interior of the cell, and there again transporters in organelles such as vacuoles [22,24]. It also depends on the ability to detoxify reactive oxygen species and enzyme inhibition caused by heavy metals. In fact, it could be expected that some of the metallophilic fungal proteins can be more robust than their counterparts in non-metallophilic fungi. During the fungal–plant interaction there are several mechanisms that could enhance metal bioremediation. Firstly, it has been reported that many fungal species produce phytohormones such as gibberellins, auxins, etc. that modify the root architecture, and this could augment the absorption surface in the soil since the fungal mycelia may create a big network of hyphae, thus taking up more metals than the lesser surface area of roots without fungal colonization. Once inside the plant, fungi could compartmentalize heavy metals in the vacuole or other membranous organelles such as lysosomes, peroxisomes, etc., thus reducing the damage to the plant cells by lowering the heavy metal concentration inside the root. Additionally, the fungus could act as a metal translocator, increasing the accumulation of heavy metals in the shoot, which is the main mechanism used by hyperaccumulator plants [22,23,24]. Finally, chelating agents such as organic acids, glutathione and metallothioneins can also play an important role in metal tolerance.

It is possible that some metallophile fungal proteins are not affected by high metal concentrations, but this remains to be explored. 

Since strain C7 was the most promising strain in terms of metal tolerance, this strain was molecularly identified. Phylogenetic analysis and long-read sequence comparisons indicated that it belongs to a strain of *Aspergillus luchuensis*, under the assumption that organisms of the same species share significant genomic coherence indices (mutational distance D ≤ 0.05 and containments ≥ 99%) [34,35]. The genome of this species has been completely sequenced, since the mold is used to produce awamori, a rice-fermented beverage very popular in Japan. An outstanding feature is that no clusters were found for the synthesis of fumosins or ochratoxins [36], which makes the use of this species safe for biotechnological applications. There is a report [37] in which the authors describe a strain of *A. luchuensis* highly tolerant to copper (417 ppm). However, it remains to be determined whether this strain is indeed *A. luchuensis*, since only morphological techniques were used for its identification and it can be easily confused with *A. kawachii* and *A. acidus* or even *A. awamori* or *A. tubingensis* [38].

*A. luchuensis* has not been thoroughly studied, but a few papers show its potential as a phenolic compound degrader [39,40], and there are several studies of this species as a producer of citric acid [41,42]. The latter characteristic could be related to its plant growth-promoting activity, since it has been shown that lowering the pH of the soil enhances phosphorous solubilization [43]. Also, citric acid can act as a chelator of metals, thus increasing its tolerance to these elements [42].

### 3.2. Plant Growth Promotion of A. luchuensis C7 and Influence of Heavy Metal Exposure on Size Characters in P. laevigata Individuals

The ability of *A. luchuensis C7* to promote plant growth was tested, and *P. laevigata* plants were clearly stimulated in plant growth by strain C7 when grown in the control substrate, although no effect was found when grown in the mine tailing substrate. However, this phenomenon was not due to metal toxicity to the plant, since plants grown on non-polluted substrate without fungal inoculation showed no statistical difference with plants grown in the mine tailing, either with or without fungal inoculation. Since the mine tailing contains similar concentrations of Cu, Zn and Pb as the ones tested in vitro, it could be possible that other element(s) present in the mine tailing (for example, Cd or As) could be toxic to the fungus or abolish its plant growth-promoting effect due to a still-unidentified mechanism. It must be remembered that *A. luchuensis* C7 was isolated from inside the root of *P. laevigata*, which was collected in the field (thus it is an endophyte fungus). However, the colonization mechanism of the roots in such toxic environments has not been thoroughly explored. The fungus may migrate to the seeds being transmitted in a vertical manner, so it is protected from other toxic metals like As or Cd since the beginning of the interaction [44]. In this work, spores obtained from in vitro cultures were added to the different substrate soils, so the fungus had to germinate in the presence of other toxic compounds (or ionic force or pH, for example) present in the polluted soil. Nevertheless, the fungus in the mine tailing substrate did have an effect on metal translocation within the plant (see below). Experiments remain to be done to explore these phenomena.

Control substrate with fungi promoted larger size characters in *P. laevigata* individuals in comparison with the rest of the treatments. This result provides evidence that *A*. *luchuensis* strain is a growth-promoting agent for *P. laevigata* individuals. Plant growth-promoting abilities of fungi can be due to a series of different mechanisms or several of them at the same time. Among them, organic acid secretion—which either dissolves or chelates several substances, making them bioavailable (in the case of nutrients, for example phosphate)—or on the contrary, immobilizing toxic elements such as heavy metals.

### 3.3. Heavy Metal Bioaccumulation and Translocation in P. laevigata Individuals

With respect to Cu translocation, as exposure to tailing substrate with fungi increased, Cu translocation from root to leaf also increased in a statistically significant manner. This finding is very interesting since the fungus is promoting an increased Cu translocation.

Copper is the active component in many fungicides that have been used in agriculture for many decades. Consequently, many fungal species have established defense mechanisms to ameliorate the toxicity of heavy metals, including copper. These mechanisms are generally based on metal immobilization through the production of intracellular and extracellular chelating compounds. Also, other studies have demonstrated that fungal strains isolated from polluted zones are capable of metal scavenging [40,45]. Fungi exhibit a high ability to immobilize toxic metals by insoluble metal oxalate formation, biosorption, or chelation onto melanin-like polymers [46]. Furthermore, due to the low substrate specificity of their degradative enzyme machinery like laccase and manganese peroxidase, fungi can perform the breakdown of different pollutants in contaminated soils.

Our results showed a positive and significant relationship between exposure time and copper TF in *P. laevigata* with fungi. In contrast, we registered higher TF in the absence of fungi in some cases; however, they did not show a significant relationship through exposure time. 

In future phytoremediation strategies, we recommend inoculating *A. luchuensis* in the substrate without metals, where *P. laevigata* individuals are established, to have plants with greater biomass and size that can absorb and bioaccumulate higher heavy metal concentrations. Thereafter, *P. laevigata* individuals will bioaccumulate more metals and in turn will translocate more metals—especially copper—from soils to roots and then to the aerial parts, as time exposure also increases. This is particularly important in arboreal species with perennial life forms, like *P. laevigata*, which is a tree species that develops naturally in mine tailings and has been reported to bioaccumulate metals such Cu. On the other hand, even when TF without fungi were higher at some exposure times, we did not document a statistically significant relationship between exposure time and ad copper TF. Thus, we recommend using this approach in phytoremediation strategies for copper contaminated soils.

In this context, a report describing an environmental isolate of an *A. piperis* strain—a species closely related to *A. luchuensis* (Figure 5)—which was tolerant to heavy metals was recently published by de Wet and Brink [47]. This strain could withstand up to 2000 ppm of lead (Pb) as measured by the agar well diffusion technique and could remove 82% of 500 ppm in liquid culture in 96 h. The strain isolated in this work proved to be metallophilic, since it could grow better than in the controls without metals in Pb at 500, 1000 and 3000 ppm, and even grew very similarly to the control without metals in 4000 ppm of Pb. Hence, probably *A. luchuensis* and *A. piperis* have developed tolerance mechanisms toward heavy metals—including copper—that enables them to play a key role in copper translocation from root to leaves, according to the results obtained in the present study. Also, in a previous study conducted by Muro-González et al. [27] using the same tailing substrate as the present study, they reported that copper showed the highest (92.2%) translocation values, which were observed in *P. laevigata* individuals. In particular, in this study species our results could be explained because copper is a trace element that can also be translocated to the aerial parts, due to its role as an important component in regulatory proteins. It participates in electron transport in chloroplasts and mitochondria of foliar cells, and it acts as a cofactor of enzymes like Cu-SOD and cytochrome oxidase. Also, it plays a part in different metabolic processes—for example, hormonal signaling, cell wall metabolism, and stress response [48]. Moreover, copper translocation levels in *P. laevigata* leaves might be one of the main Cu detoxification mechanisms, like other metals such as Mn, Pb, Zn [49].

The present findings could explain, in part, the high translocation values observed in previous studies with this metal accumulator plant, because the *A. luchuensis* strain found in this study establishes naturally on *P. laevigata* roots, favoring copper bioaccumulation and translocation. 

Another interesting observation was that metal translocation values for Pb, Cu and Zn in *P. laevigata* individuals growing on tailing substrate with fungi were greater throughout exposure time in comparison with metal translocation values for individuals growing in tailing substrate without fungi (Table 5). This finding is an additional element that supports the results obtained, in which *A. luchuensis* favors not only copper translocation, but it may also permit other metal translocation processes to occur.

Although some living organisms have been proven useful for remediating metal-contaminated soils [49], plant symbioses have been scarcely studied concerning their use in improving phytoremediation processes. Therefore, the use of this strain would be an important tool in remediating Cu-contaminated soils. Finally, in this report we propose this new *A. luchuensis* strain, which with its endophytic character and plant growth-promotion activity, along with its high metal tolerance and its ability to increase copper translocation to foliar plant tissue, could be a novel, effective and sustainable bioremediation strategy for copper-polluted soils.

## 4. Materials and Methods

### 4.1. Plant Species

*Prosopis laevigata* is a tree commonly known as mezquite. It presents a broad range of geographical distribution in Mexico. *P. laevigata* includes large trees of up to 13 m in height and trunk diameter of 80 cm. The flowering period occurs from February to May and fruits are produced from June to July. It sheds its leaves in winter and grows naturally and abundantly in Huautla, Morelos mine tailings.

### 4.2. P. laevigata Sampling and Root Collection

Four healthy trees of *P. laevigata* were chosen, between 6 and 8 m in height from the main Huautla mine tailing. Herbarium samples of the individuals were identified as *Prosopis laevigata* Humb. & Bonpl. ex Willd. at the HUMO herbarium (Herbarium of the Research Center in Biodiversity and Conservation, Autonomous University of Morelos State, Mexico). From each of the four individuals, adventitious roots were cut in 10 cm long pieces and subsequently root pieces were transported to the laboratory inside an ice box under darkness at 4 °C in 25 mL conical tubes until use.

### 4.3. Fungal Endophyte Isolation

The roots of *P. laevigata* were washed with tap water for ten minutes and then again washed for five minutes with sterile double-distilled water. Subsequently, inside a laminar flow hood, the roots were submerged for three minutes in a 70 % *v*/*v* ethanol solution, then were immersed in a solution of 4 % NaOCl supplemented with Tween 80^®^ (0.1 % *v*/*v*) for five more minutes. Finally, the roots were washed with sterile double-distilled water for one minute and dried.

Once the roots were superficially disinfected, pieces of 4 × 0.5 cm or 1.5 × 0.5 cm were cut and sown in Petri dishes with Potato Dextrose (PDA, Difco^®^ Difco, Baltimore, MD, USA) in a pH 5.5 culture medium. To inhibit the growth of endophytic bacteria, the medium was supplemented with 50 μg/mL of streptomycin (Str) and 50 μg/mL of amoxicillin (Am). The incubation conditions were the following: fixed incubator, incubation temperature of the plates was 28–30 °C for 40 days, in darkness. Every two days the Petri dishes were revised and, when visible, the tips of emerging hyphae from the roots were isolated. Three passes for each isolate were given to ensure that no mixed populations were carried along. Ten different morphotypes were easily identified and isolated.

### 4.4. Minimum Inhibitory Concentration (MIC) as Inhibition of Radial Growth of the Fungal Isolates

To determine the MIC of the isolated endophytic fungal strains, they were cultured in PDA medium added with different concentrations of copper sulfate pentahydrate (CuSO_4_·5H_2_O) (50, 100 and 200 ppm); lead nitrate (Pb (NO_3_)_2_ (600, 1800, 3000 and 4000 ppm) and zinc sulfate heptahydrate (ZnSO_4_·7H_2_O) (300, 950 and 1600 ppm), according to the bioavailability concentrations found in the mine tailing [28].

We determined the MIC (the concentration in which no growth was observed) by inoculating 8 mm Ø agar disks obtained with a boring drill from plates of freshly grown reactivated fungi in the center of the metal-containing Petri dish. Radial growth was evaluated for the concentrations mentioned above. Controls were accomplished using the same procedure in metal-free PDA medium. The plates were incubated at 30 °C for eight days and every two days the radial growth of the fungi was measured. All concentrations for each metal were evaluated in triplicate, including the control.

Once we had the MIC for each metal and fungal strain, we evaluated the growth of fungi in the presence of the three metals (Cu, Zn and Pb) in the same medium. The concentrations that we used were 50 ppm of Cu, 300 ppm of Zn and 600 ppm of Pb (Treatment 1); 100 ppm of Cu, 950 ppm of Zn and 1800 ppm of Pb (Treatment 2); 200 ppm of Cu, 1600 ppm of Zn and 4000 ppm of Pb (Treatment 3). We inoculated an 8 mm Ø disk with the mycelium of the fungus in each of the media, and evaluated the radial growth in the same way as the aforementioned procedure. 

### 4.5. Growth Rate and Inhibition Percentage of Endophytic Fungal Strains

For each of the strains in each of the concentrations of Cu, Zn and Pb indicated by the MIC, we calculated the growth rate, as the means of an average of the values obtained (per triplicate) from the measurement of radial growth through time using a scatter plot of data to calculate the slope, the value of which corresponds to the growth rate.

For the percentage of inhibition, the following formula was used:

Percent Inhibition = 100 − (TCM ∗ 100/TCC)

where:

TCM: Growth rate of the fungus to be analyzed in the presence of the heavy metal.

TCC: Growth rate of the control fungus (without metals).

### 4.6. Identification of Endophytic Fungal Strain C7

The morphological analysis was carried out by cultivating the strain in PDA medium and staining seven-day-old mycelia with lactophenol blue to observe the asexual reproduction structures such as conidiophores, conidiogenous cells and conidia under the microscope. Lactophenol blue stain is a simple stain that is based on the affinity of the dye for cellular components, particularly fungal structures. Phenol destroys the accompanying microbiota if there are contaminants in the sample, lactic acid preserves fungal structures due to an osmotic gradient between the inside and outside of the structure, and cotton blue can adhere to hyphae and conidia, making it possible to observe them at the 40X and 100X objectives. The observed images were contrasted with images found on the internet for possible identification.

To ensure the correct identification of C7, we obtained a preliminary draft of the genome by low deep long-read sequencing (≤40X) (MinION, Oxford Nanopore Technologies) platform from the Instituto de Biotecnología, Universidad Nacional Autónoma de México. The quality of the reads was assessed with Filtlong v0.2.1 (https://github.com/rrwick/Filtlong, accessed on 21 March 2022) and Porechop v.0.2.4 tools (https://github.com/rrwick/Porechop, accessed on 21 March 2022). We used Minimap2 [50] to select the reads set that map to *A. piperis* and *A. luchuensis* representative genomes. We obtained a subset of 156,958 mapped reads which was used as seed to search for homologous sequences to the calmodulin (calm) and beta-tubulin (tub) genes, taking as queries the NBRC Culture Catalogue Sequece IDs: IF00428104 and IF00428103. 28S coding sequence was directly predicted with Barrnap v0.9 (https://github.com/tseemann/barrnap, accessed on 21 March 2022) option: --kingdom. Sequences producing significant alignments were selected based on Score (bits) criteria, Expect and Identities. With the predicted sequences (calm, tub, 28S) for *Aspergillus* sp. C7, we explored the space of orthologous in the NCBI type material database and selected 16 closest non-redundant hits for each marker. Each set of markers was aligned independently with MAFFT aligner v7.453 [51] and trimmed with trimAl v1.2 -gappyout option [52]; the alignments were concatenated and a maximum likelihood phylogeny estimated within Seaview v4.5.4 [53] under TN93 model [54]. The model was selected according to the best Akaike Information Criterion [55]. Alternatively, the raw long-reads subset of strain C7 was compared against a custom fungal minhashed database to assess containment and genomic distance using the software Mash -function mash screen- [29].

### 4.7. Seed Collection and Germination of P. laevigata for Plant Growth Promotion and Metal Accumulation Experiments

*P. laevigata* seeds were collected from individuals established in Quilamula, Morelos control site. Mature and healthy fruits that presented complete pods were randomly selected, with no apparent damage by fungi or borer insects. Twenty individuals were randomly selected, and 20% of their seeds were collected [56]. The seeds were transported to the laboratory, where they were cleaned and selected, removing the seeds parasitized by insects. Finally, they underwent a mechanical scarification process to obtain a more efficient germination. For germination, 90 seeds were placed in three Petri dishes (30 seeds per box) on cotton wool moistened with distilled water. Once seeds were germinated, they were placed in trays with peat moss substrate until the plant reached a size between 4 and 5 cm. This procedure was carried out for seven days at 32 °C. Later, 90 seedlings were transplanted into bags with treatment substrates (45 individuals in each treatment).

### 4.8. Tailing and Reference Substrates Sampling for Plant Growth Experiments

*Tailing substrate*: To carry out the experiment under greenhouse conditions, tailing substrate was collected through a superficial and random sampling in the two most preserved tailings located in Huautla, Morelos. After that, the samples collected from both tailings were homogenized with a shovel, removing stones and root debris to obtain the substrate that was used to fill bags with a capacity of 4 L to transplant the *P. laevigata* seedlings.

*Reference substrate*: The substrate was collected through a superficial and random sampled in Quilamula, Morelos, where there are no reports of heavy metal exposure from mining activity. This substrate was sieved to 3 mm to obtain a similar texture to the mining substrate, and it was used to transplant the reference seedlings of *P. laevigata*.

### 4.9. Growing of P. laevigata in Control and Tailing Substrates with or without Fungal Inoculation

*P. laevigata* seedlings were transplanted to a previously sterilized tailing substrate homogenate and control substrates. Fifty replicates were seeded, for a total of six measurements per month in a period of 6 months for each substrate. Given the data obtained from previous experiments, we decided to test only *A. luchuensis* C7 for the plant experiments. We used as control *P. laevigata* plants grown in sterilized tailing and control substrates without inoculating the endophytic fungus.

### 4.10. Inoculum Preparation for P. laevigata

For each isolate, we carried out a pre-inoculum by taking a 0.5 diameter × 0.8 cm thick disk and placing each one on a Petri dish with PDA pH 5.5 medium, then incubating in darkness at 28–30 °C until the necessary biomass of endophytic fungus was obtained.

From freshly grown PDA dishes, spores from *A. luchuensis* C7 were collected in 1 mL of 0.9 % sterile saline solution to obtain a spore suspension (6 repetitions). In a 96-well plate, dilutions from 10-1 to 10-3 were prepared by placing 90 µL of sterile distilled water and 10 µL of the suspension in each well. To perform the spore count, 10 µL of the 10-2 dilution for each repetition was placed in a Neubauer chamber and spores were counted in each quadrant. With these data, the calculation to determine the necessary µL of inoculum to have 106 spores per sample was performed (8.8 µL for the control substrate and 8.5 µL for the tailing substrate).

### 4.11. Plant Growth Promotion

Plant growth promotion by *A. luchuensis* C7 was determined in both substrates (control and tailing). Each month, six individuals from each treatment (control substrate with fungus and without fungus, tailing substrate with fungus and without fungus) were collected to measure the number of leaves (#), total fresh weight and root dry weight (g), fresh and dry weight of leaves (g) and height of the plant (cm).

### 4.12. Concentration of Heavy Metals in Roots and Leaves of P. laevigata during Growth in the Presence or Absence of A. luchuensis C7, in Tailing and Control Substrates

A total of 72 samples (six roots and six leaves per treatment) of plants grown in the tailing substrate were analyzed to determine the metal concentrations (Pb, Cu, Zn) during a six-month period. The root tissues were first washed with tap water and subsequently with distilled water to remove the residue from the substrates. All the tissues were taken to a drying oven at 60 °C until they reached a constant weight. Each plant tissue (0.25 g) was placed and pulverized in a container previously washed with HNO3. The samples were subjected to acid digestion using 10 mL of HNO3 (70%) in closed Teflon pumps. Each sample was diluted in distilled water (up to 50 mL per sample) and filtered; this solution was stored at 4 °C until analyzed. The metals were then analyzed by Atomic Absorption Spectrophotometry (GBC-908-AA, Scientific equipment), calibrating the spectrophotometer with standard solutions containing known concentrations of each of the analyzed elements. The concentration of the three metals analyzed was determined by calibration curves obtained using internal standard solutions of pure metal ions (Ultra Scientific). The standard calibration curves showed correlation coefficients (R^2^) between 0.99 and 1. The minimum detection limits (mg/L) according to the manufacturer are: Pb (0.01), Cu (0.001), and Zn (0.0005). For each measurement, the average value of three replicates was reported.

### 4.13. Statistical Analysis

One-way ANOVA was conducted to determine the effect of treatment (substrate: reference with fungi, control without fungi, tailing with fungi, and tailing without fungi) on each size character measured. Count characters were transformed as [(x) 1/2 + 0.5]. To test for normality and homogeneity of variance, Shapiro-Wilk and Levene tests were employed. Significant mean differences between treatment (substrate) were determined with a post-hoc Tukey multiple range test (*p* < 0.05) [57]. 

Discriminant function analysis (DFA) was carried out using all size variables. We performed a separate DFA for exposure time (six months). The purpose of this analysis was to determine the most useful size characters to discriminate between treatments and to visually assess the separation of individuals into groups. We established the type of treatment as the predictor variable.

Additionally, we performed a simple regression analysis between exposure time to substrate and size characters of *P. laevigata* individuals under greenhouse conditions.

The capacity of *P. laevigata* to phytoextract heavy metals was evaluated using the translocation factor (TF), which measures the efficiency of the plant in the transportation of metals from the root to the aerial parts [58]. This index is calculated as follows:

TF = Cfoliar/Croot

where Cfoliar is the concentration of the metal in the leaf tissue and Croot is the concentration of the metal in the root tissue. It has been reported that if a plant has TF values > 1, the species is considered an accumulator of the analyzed metal [58,59]. Finally, a simple regression analysis was conducted between exposure time and heavy metal translocation values (TF) in *P. laevigata* exposed to substrate with fungi and without fungi.

Statistical analyses were performed with Past 4.01 [60] and STATISTICA software version 8.0 [61].

## 5. Conclusions

A novel strain of *Aspergillus* (strain C7) closest to *A. luchuensis,* isolated from the metal hyperaccumulator plant *P. laevigata,* was determined to be a metallophile and presented a marked tolerance to high concentrations of Cu, Zn and Pb. Also, the fungus promoted larger size characters in *P. laevigata* individuals, demonstrating that the C7 strain is a growth-promoting agent for *P. laevigata* individuals. Moreover, the fungus favored the translocation of metals from roots to leaves in *P. laevigata*, promoting an increased Cu translocation. Overall, we conclude that this new *A. luchuensis* strain has endophytic character, plant growth-promotion activity, high metal tolerance, and an ability to increase copper translocation. Hence, we propose it as a novel, effective and sustainable bioremediation strategy for copper-polluted soils.

## Figures and Tables

**Table 1:** Endophyte growth test with heavy metals (Cu, Zn, Pb) under two extreme concentrations for each element.

	Metals (ppm)					
Strain	Cu		Zn		Pb	
	50	200	300	1600	600	4000
C1	+++	+++	+++	-	+++	++
C2	+++	+++	+++	-	+++	++
C3	++	++	++	+	++	++
C4	++	++	++	-	++	++
C5	+++	+++	+++	++	+++	++
C6	+++	++	++	+	++	-
C7	+++	+++	+++	-	+++	+++
C8	+++	+++	+++	-	+++	+++
C9	+	+	+	-	++	-
C10	+++	+++	+++	+	+++	+

The + signs indicate diameter of the colonies: (-) = no growth (0 cm); (+) = little growth (0.1–2 cm); (++) = good growth (2.1–5 cm); (+++) = excellent growth (5.1–8 cm).

**Table 2:** Closest neighbors for *Aspergillus luchuensis* strain C7 based on 28S, calmodulin and β-tubulin markers blast comparison. Only three representative hits are shown per marker.

Accession	Description	Identity (%)	Score	Cover (%)	E Value
XR_005976915.1	*Aspergillus luchuensis* IFO 4304 28S	99.81	6752	100	0
XR_005951996.1	*Aspergillus flavus* NRRL3357 28S	97.83	6342	100	0
JN642222.1	*Penicillium solitum* 20-01 28S	96.22	6006	99	0
LC573714.1	*Aspergillus luchuensis* NBRC 6086 calmodulin (partial cds)	96.79	1230	100	0
EF661152.1	*Aspergillus tubingensis* NRRL 4750 calmodulin (partial cds)	96.73	1203	97	0
KF900176.1	*Penicillium dimorphosporum* NRRL 5207 calmodulin (partial cds)	84.07	398	56	2 × 10^−7^
LC573653.2	*Aspergillus luchuensis* NBRC:4033 beta-tubulin (partial cds)	95.81	1563	100	0
LC573644.1	*Aspergillus foetidus* NBRC:4338 beta-tubulin (partial cds)	95.81	1563	100	0
AY846880.1	*Penicillium paxilli* CBS:360.48 beta-tubulin (complete cds)	86.50	911	86	0

**Table 3:** Genomic coherence for a set of long-reads of strain C7 against representative closest fungal genomes.

Accession	Description	Mutational Distance (*D*)	Shared Hashes	Mash Screen Identity	Shared Hashes
GCA_016861625.1	*Aspergillus luchuensis*	0.0375603	294/1000	0.984152	715/1000
GCA_003184755.1	*Aspergillus piperis*	0.0533217	195/1000	0.97022	530/1000
GCA_003184625.1	*Aspergillus neoniger*	0.0747985	116/1000	0.95188	355/1000
GCA_003184535.1	*Aspergillus eucalypticola*	0.0740698	118/1000	0.951367	351/1000

**Table 4:** Average values ± standard error and one-way ANOVA results to evaluate the effect of treatment (control with fungi, control without fungi, tailing with fungi and tailing without fungi) on size characters of *Prosopis laevigata* growing under greenhouse conditions. Different letters show significant differences in individuals growing under different treatments (Tukey *p* < 0.05). * *p* < 0.05, ** *p* < 0.01, *** *p* < 0.001, n.s. = not significant differences.

	Characters						
Treatment (Substrate)	Dry Root Biomass	Dry Leaf Biomass	Height	Number of Leaves		Wet Root Biomass	Wet Leaf Biomass
	Month 1						
Control with fungi	0.050 ± 0.000 a	0.075 ± 0.011 a	11.462 ± 0.329 a	18.667 ± 0.615 a		0.375 ± 0.042 a	0.158 ± 0.008 a
Control without fungi	0.042 ± 0.014 a	0.047 ± 0.012 ac	9.167 ± 0.710 b	16.000 ± 1.461 ac		0.283 ± 0.053 a	0.047 ± 0.012 b
Tailing with fungi	0.005 ± 0.002 b	0.011 ± 0.002 b	6.000 ± 0.485 c	9.333 ± 0.955 b		0.034 ± 0.011 b	0.033 ± 0.005 b
Tailing without fungi	0.007 ± 0.001 b	0.018 ± 0.002 bc	6.700 ± 0.331 c	12.100 ± 1.915 bc		0.062 ± 0.009 b	0.054 ± 0.006 b
ANOVA	*F*_3,20_ = 11.169 ***	*F*_3,20_ = 12.279 ***	*F*_3,20_ = 25.502 ***	*F*_3,20_ = 9.697 ***		*F*_3,20_ = 23.379 ***	*F*_3,20_ = 48.903 ***
	Month 2						
Control with fungi	0.199 ± 0.030 a	0.134 ± 0.025 a	15.617 ± 1.685 a	28.833 ± 0.038 a		0.859 ± 0.133 a	0.317 ± 0.070 a
Control without fungi	0.064 ± 0.016 b	0.033 ± 0.007 b	8.000 ± 0.639 b	11.167 ± 1.400 b		0.343 ± 0.092 b	0.065 ± 0.015 b
Tailing with fungi	0.045 ± 0.015 b	0.024 ± 0.007 b	8.017 ± 0.670 b	13.833 ± 2.120 b		0.334 ± 0.099 b	0.060 ± 0.021 b
Tailing without fungi	0.036 ± 0.009 b	0.025 ± 0.005 b	7.917 ± 0.559 b	14.500 ± 2.579 b		0.258 ± 0.065 b	0.059 ± 0.013 b
ANOVA	*F*_3,20_ = 15.716 ***	*F*_3,20_ = 13.350 ***	*F*_3,20_ = 14.553 ***	*F*_3,20_ = 8.735 ***		*F*_3,20_ = 7.579 **	*F*_3,20_ = 11.419 ***
	Month 3						
Control with fungi	0.416 ± 0.059 a	0.119 ± 0.025 a	14.000 ± 0.899 a	35.500 ± 3.030 a		2.159 ± 0.272 a	0.294 ± 0.772 a
Control without fungi	0.152 ± 0.040 b	0.046 ± 0.013 b	7.883 ± 0.558 b	24.000 ± 6.648 ab		0.837 ± 0.170 b	0.109 ± 0.034 b
Tailing with fungi	0.094 ± 0.028 b	0.018 ± 0.004 b	7.367 ± 0.801 b	12.167 ± 3.038 b		0.414 ± 0.122 b	0.034 ± 0.009 b
Tailing without fungi	0.092 ± 0.029 b	0.020 ± 0.009 b	8.083 ± 0.961 b	9.667 ± 2.348 b		0.266 ± 0.026 b	0.038 ± 0.022 b
ANOVA	*F*_3,20_ = 14.266 ***	*F*_3,20_ = 9.847 ***	*F*_3,20_ = 14.552 ***	*F*_3,20_ = 7.619 **		*F*_3,20_ = 24.999 **	*F*_3,20_ = 7.694 ***
	Month 4						
Control with fungi	0.248 ± 0.033 a	0.087 ± 0.016 a	10.083 ± 1.228 a	38.833 ± 7.507 a		1.381 ± 0.095 a	0.144 ± 0.043 a
Control without fungi	0.145 ± 0.030 ab	0.040 ± 0.005 b	8.317 ± 0.599 a	25.167 ± 3.945 ab		0.943 ± 0.145 ab	0.066 ± 0.009 ab
Tailing with fungi	0.139 ± 0.031 ab	0.024 ± 0.008 b	9.267 ± 0.847 a	14.000 ± 3.055 b		0.550 ± 0.136 bc	0.040 ± 0.016 b
Tailing without fungi	0.081 ± 0.016 b	0.020 ± 0.004 b	6.717 ± 0.668 a	18.333 ± 3.148 b		0.398 ± 0.089 c	0.029 ± 0.006 b
ANOVA	*F*_3,20_ = 6.161 **	*F*_3,20_ = 10.138 ***	*F*_3,20_ = 2.760 n.s.	*F*_3,20_ = 5.170 **		*F*_3,20_ = 13.713 **	*F*_3,20_ = 4.693 *
	Month 5						
Control with fungi	0.610 ± 0.77 a	0.136 ± 0.021 a	16.217 ± 2.371 a	47.500 ± 7.065 a		3.389 ± 0.431 a	0.287 ± 0.047 a
Control without fungi	0.149 ± 0.023 b	0.034 ± 0.012 b	8.533 ± 1.102 b	24.333 ± 6.922 b		1.015 ± 0.192 b	0.0625 ± 0.227 b
Tailing with fungi	0.221 ± 0.141 b	0.019 ± 0.004 b	7.183 ± 0.379 b	15.000 ± 3.225 b		0.412 ± 0.054 b	0.031 ± 0.008 b
Tailing without fungi	0.153 ± 0.041 b	0.026 ± 0.010 b	8.933 ± 1.438 b	14.000 ± 3.225 b		0.675 ± 0.173 b	0.041 ± 0.015 b
ANOVA	*F*_3,20_ = 6.930 **	*F*_3,20_ = 17.404 ***	*F*_3,20_ = 7.323 **	*F*_3,20_ = 8.177 ***		*F*_3,20_ = 29.255 ***	*F*_3,20_ = 19.623 ***
	Month 6						
Control with fungi	0.633 ± 0.105 a	0.195 ± 0.026 a	16.517 ± 1.377 a	98.833 ± 14.827 a		2.625 ± 0.369 a	0.367 ± 0.042 a
Control without fungi	0.237 ± 0.034 b	0.071 ± 0.010 b	10.550 ± 0.617 b	42.667 ± 8.838 b		1.158 ± 0.171 b	0.126 ± 0.025 b
Tailing with fungi	0.055 ± 0.003 b	0.032 ± 0.013 b	7.240 ± 0.430 b	14.800 ± 1.077 b		0.549 ± 0.098 b	0.040 ± 0.006 b
Tailing without fungi	0.037 ± 0.012 b	0.022 ± 0.009 b	6.600 ± 0.678 b	20.500 ± 6.840 b		0.416 ± 0.257 b	0.045 ± 0.019 b
ANOVA	*F*_3,20_ = 24.882 ***	*F*_3,20_ = 24.328 ***	*F*_3,20_ = 28.186 ***	*F*_3,20_ = 17.012 ***		*F*_3,20_ = 16.948 ***	*F*_3,20_ = 33.850 ***

**Table 5:** Mean (±standard error) and simple regression analysis between exposure time and translocation values (FT) of heavy metals in *Prosopis laevigata* growing under greenhouse conditions. Bold letters denote FT values greater than one and denote significant differences in regression analysis.

Time	Lead (Pb)		Copper (Cu)		Zinc (Zn)	
(Months)	With Fungi	Without Fungi	With Fungi	Without Fungi	With Fungi	Without Fungi
1	**4.2 ± 0.75**	0.3 ± 0.02	0.1 ± 0.04	0.3 ± 0.04	0.0 ± 0.02	0.0 ± 0.00
2	**2.6 ± 0.34**	**1.3 ± 0.44**	0.9 ± 0.19	**1.2 ± 0.33**	0.3 ± 0.00	0.5 ± 0.06
3	**8.1 ± 1.02**	**10.1 ± 1.98**	**4.0 ± 0.20**	**7.3 ± 0.65**	**6.2 ± 0.88**	**4.1 ± 0.81**
4	**1.2 ± 0.28**	**2.6 ± 0.35**	**1.7 ± 0.22**	**3.3 ± 0.75**	**1.4 ± 0.33**	**3.6 ± 0.89**
5	**4.0 ± 1.13**	**7.3 ± 1.18**	**8.4 ± 0.69**	**8.3 ± 1.24**	**2.6 ± 0.61**	**6.1 ± 0.40**
6	**5.3 ± 1.01**	**1.9 ± 0.42**	**16.9 ± 3.65**	**5.9 ± 0.92**	**10.0 ± 1.25**	0.9 ± 0.27
Mean	4.2 ± 0.48	3.9 ± 0.70	5.3 ± 1.14	4.4 **± 0.58**	3.4 ± 0.66	2.5 ± 0.43
	*r* = 0.075,	*r* = 0.450,	*r* = 0.870,	*r* = 0.737,	*r* = 0.710,	*r* = 0.455,
Regression	*r*^2^ = 0.243,	*r*^2^ = 0.202,	*r*^2^ = 0.695,	*r*^2^ = 0.429,	*r*^2^ = 0.340,	*r*^2^ = 0.008,
	*p* = 0.888	*p* = 0.370	***p* = 0.024**	*p* = 0.09	*p* = 0.114	*p* = 0.365

## Data Availability

Data recorded in the current study are available in all tables of the manuscript.

## Supplementary Materials

The following supporting information can be downloaded at: https://www.mdpi.com/article/10.3390/plants12061338/s1, Table S1. Discriminant function analysis for size character variation of P. laevigata in different exposure times (six months) growing under greenhouse conditions. The bold letters show the variable that most contribute to the DFA ordination. Table S2. Average values (± standard deviation) of heavy metal concentration (mg Kg^−1^) in root and leaf of Prosopis laevigata growing on tailing substrate under greenhouse conditions. Table S3. Simple regression analysis between exposure time and size characters of Prosopis laevigata individuals growing under greenhouse conditions. The bold letters denote significant differences. Table S4. Simple regression analysis between exposure time and heavy metal bioaccumulation in root and leaf of Prosopis laevigata growing under greenhouse conditions. The bold letters denote significant differences.
