# Supplementary material for: Aspergillus luchuensis, an Endophyte Fungus from the Metal Hyperaccumulator Plant Prosopis laevigata, Promotes Its Growth and Increases Metal Translocation
Source: Plants (Basel). 2023 Mar 16;12(6):1338. doi: 10.3390/plants12061338 (PMC10053625; doi:10.3390/plants12061338)
Supplement: Supplementary file 1 [file plants-12-01338-s001.zip › Supplementary Tables S1 to S4.pdf]

Supplementary Table S1. Discriminant function analysis for size character variation of *P. laevigata* in different exposure times (six months) growing under greenhouse conditions. The bold letters show the variable that most contribute to the DFA ordination.

Variable	DF1	DF2	DF1	DF2
	Month 1		Month 2	
Dry root biomass	**-53.85**	-5.64	20.99	5.04
Dry leaf biomass	-28.32	**31.37**	**141.05**	**154.23**
Height	-0.59	0.67	-0.16	-0.75
Number of leaves	-0.05	0.01	-0.05	-0.17
Wet root biomass	5.47	5.89	-2.78	1.85
Wet leaf biomass	**105.24**	**-36.98**	**-41.35**	**-41.90**
Eigenvalue	14.87	3.68	4.30	0.70
% Variation	79.88	19.78	84.72	13.75
Significance	< 0.001	< 0.001	< 0.001	< 0.001
	Month 3		Month 4	
Dry root biomass	-27.29	**-13.50**	4.80	27.16
Dry leaf biomass	**144.34**	**29.12**	**17.31**	**112.04**
Height	0.38	-0.36	**-0.25**	0.21
Number of leaves	0.06	0.06	0.00	-0.05
Wet root biomass	5.94	2.75	2.59	-6.95
Wet leaf biomass	**-53.54**	-10.50	1.18	**-35.76**
Eigenvalue	8.43	0.49	2.68	1.01
% Variation	94.24	5.53	68.69	25.74
Significance	< 0.001	< 0.001	< 0.001	< 0.001
	Month 5		Month 6	
Dry root biomass	4.28	1.73	0.26	5.11
Dry leaf biomass	**-6.17**	**23.36**	**-24.16**	**-106.77**
Height	0.14	-0.21	-0.14	0.12
Number of leaves	0.01	-0.06	0.02	0.01
Wet root biomass	-0.06	**-0.69**	1.91	-2.06
Wet leaf biomass	**6.94**	11.98	**11.86**	**57.12**
Eigenvalue	5.90	0.29	5.57	0.22
% Variation	93.99	4.61	95.17	3.67
Significance	< 0.001	< 0.001	< 0.001	< 0.001

Supplementary Table S2. Average values (± standard deviation) of heavy metal concentration (mg Kg-1) in root and leaf of *Prosopis laevigata* growing on tailing substrate under greenhouse conditions.

Metal	Time	Tailing			
		With fungi		Without fungi	
DL (mg/L)	(month)	root	leaf	root	leaf
Lead Pb (0.01)	1	36.35±3.35	150.87±67.05	12.39±3.43	4.05±1.19
	2	92.10±33.26	239.48±47.85	180.20±44.46	228.38±61.73
	3	34.58±9.92	281.49±86.28	30.84±9.93	311.40±97.66
	4	28.78±13.23	35.15±6.84	15.47±6.13	40.10±12.21
	5	17.21±7.09	69.45±11.81	4.35±2.91	30.46±33.63
	6	20.95±5.40	111.89±42.01	52.78±8.49	98.61±11.21
Copper Cu (0.001)	1	24.20±7.79	2.61±1.31	7.12±3.85	2.17±0.82
	2	3.39±1.29	3.14±0.27	4.47±2.98	5.18±2.57
	3	1.85±0.92	7.32±3.28	0.82±0.28	5.98±2.64
	4	5.47±3.21	9.34±3.42	6.12±1.08	19.88±5.97
	5	1.21±0.29	10.20±3.20	1.72±1.44	14.19±3.75
	6	2.02±0.99	34.18±9.04	7.22±2.26	42.91±14.75
Zinc Zn (0.0005)	1	3857.24±786.27	240.08±78.11	2457.04±841.54	4.30±0.904
	2	21.87±7.28	7.37±3.17	31.64±15.18	15.66±6.51
	3	15.13±3.65	93.84±36.74	12.89±4.04	52.33±7.40
	4	10.60±4.94	15.08±5.63	25.59±7.73	85.61±49.18
	5	16.70±7.25	43.90±13.96	25.86±11.44	158.78±42.97
	6	13.03±5.87	130.02±38.69	76.28±14.96	65.33±14.74

Supplementary Table S3. Simple regression analysis between exposure time and size characters of *Prosopis laevigata* individuals growing under greenhouse conditions. The bold letters denote significant differences.

Treatment (substrate)							
Control				Tailing			
with fungi		without fungi		with fungi		without fungi	
Dry root biomass							
r = 0.765<br>r^2 = 0.573<br>**P = 0.0000**	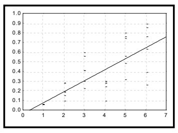	r = 0.667<br>r^2 = 0.429<br>**P = 0.0000**	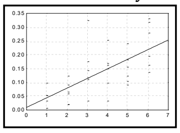	r = 0.354<br>r^2 = 0.099<br>**P = 0.034**	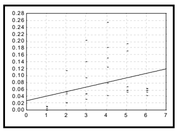	r = 0.436<br>r^2 = 0.166<br>**P = 0.008**	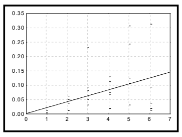
Dry leaf biomass							
r = 0.422<br>r^2 = 0.153<br>**P = 0.01**	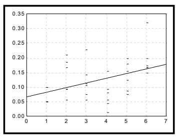	r = 0.226<br>r^2 = 0.022<br>P = 0.186	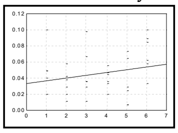	r = 0.245<br>r^2 = 0.032<br>P = 0.149	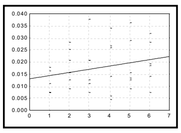	r = -0.086<br>r^2 = 0.021<br>P = 0.617	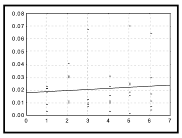
Height							
r = 0.340<br>r^2 = 0.090<br>**P = 0.042**	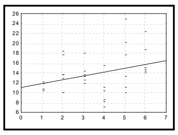	r = -0.234<br>r^2 = 0.027<br>P = 0.169	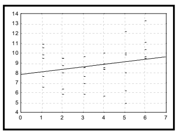	r = 0.227<br>r^2 = 0.023<br>P = 0.184	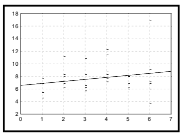	r = 0.095<br>r^2 = 0.020<br>P = 0.580	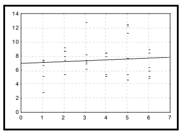
Number of leaves							
r = 0.734<br>r^2 = 0.524<br>**P = 0.0000**	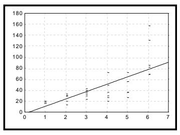	r = 0.533<br>r^2 = 0.263<br>**P = 0.0008**	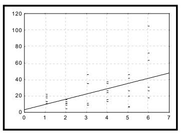	r = 0.250<br>r^2 = 0.035<br>P = 0.141	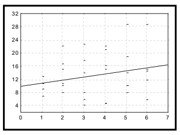	r = 0.294<br>r^2 = 0.059<br>P = 0.082	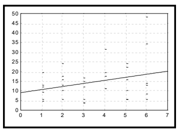
Wet root biomass							
r = 0.736<br>r^2 = 0.528<br>**P = 0.0000**	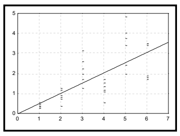	r = 0.665<br>r^2 = 0.426<br>**P = 0.0000**	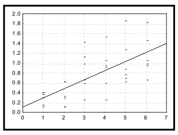	r = -0.354<br>r^2 = 0.100<br>**P = 0.033**	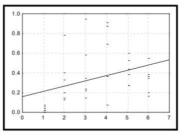	r = 0.425<br>r^2 = 0.156<br>**P = 0.009**	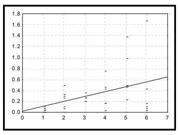
Wet leaf biomass							
r = 0.354<br>r^2 = 0.099<br>**P = 0.034**	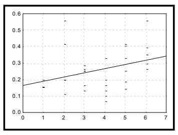	r = 0.397<br>r^2 = 0.132<br>**P = 0.0167**	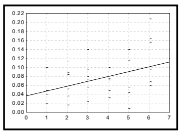	r = -0.060<br>r^2 = 0.025<br>P = 0.732	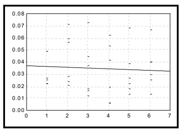	r = -0.110<br>r^2 = 0.017<br>P = 0.533	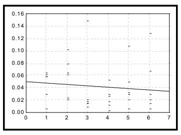

Supplementary Table S4. Simple regression analysis between exposure time and heavy metal bioaccumulation in root and leaf of *Prosopis laevigata* growing under greenhouse conditions. The bold letters denote significant differences.

Metal	Treatment (substrate)			
	Leaf		Root	
	with fungi	without fungi	with fungi	without fungi
Lead (Pb)	r= -0.53<br>r^2= 0.095<br>P= 0.284<br>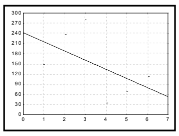	r= -0.07<br>r^2= 0.243<br>P= 0.895<br>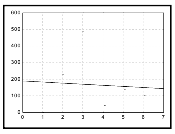	r = -0.60<br>r^2= 0.200<br>P= 0.207<br>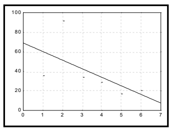	r= -0.27<br>r^2= 0.157<br>P= 0.601<br>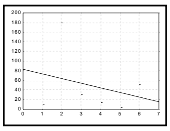
Copper (Cu)	r= 0.826<br>r^2= 0.603<br>**P= 0.043**<br>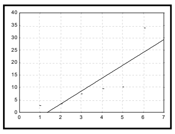	r= -0.690<br>r^2= 0.338<br>P= 0.132<br>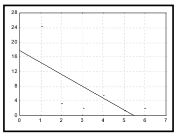	r= - 0.864<br>r^2= 0.682<br>**P= 0.026**<br>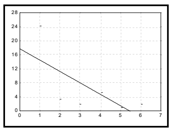	r = -0.030<br>r^2= 0.249<br>P = 0.953<br>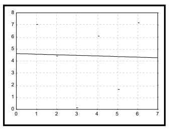
Zinc (Zn)	r= -0.320<br>r^2= 0.125<br>P= 0.542<br>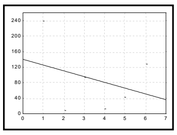	r= 0.737<br>r^2= 0.430<br>P= 0.094<br>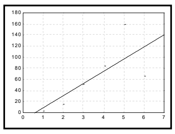	r = -0.650<br>r^2= 0.285<br>P = 0.158<br>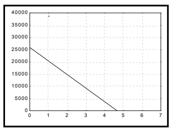	r = -0.640<br>r^2 = 0.267<br>P = 0.168<br>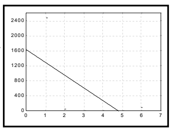
